# Identification of neoantigens derived from alternative splicing and RNA modification

**DOI:** 10.5808/GI.2019.17.3.e23

**Published:** 2019-08-22

**Authors:** Jiyeon Park, Yeun-Jun Chung

**Affiliations:** 1 Precision Medicine Research Center, College of Medicine, The Catholic University of Korea, Seoul 06591, Korea; 2 Integrated Research Center for Genome Polymorphism, College of Medicine, The Catholic University of Korea, Seoul 06591, Korea; 3 Departments of Microbiology, College of Medicine, The Catholic University of Korea, Seoul 06591, Korea

**Keywords:** alternative splicing, neoantigen, RNA editing

## Abstract

The acquisition of somatic mutations is the most common event in cancer. Neoantigens expressed from genes with mutations acquired during carcinogenesis can be tumor-specific. Since the immune system recognizes tumor-specific peptides, they are potential targets for personalized neoantigen-based immunotherapy. However, the discovery of druggable neoantigens remains challenging, suggesting that a deeper understanding of the mechanism of neoantigen generation and better strategies to identify them will be required to realize the promise of neoantigen-based immunotherapy. Alternative splicing and RNA editing events are emerging mechanisms leading to neoantigen production. In this review, we outline recent work involving the large-scale screening of neoantigens produced by alternative splicing and RNA editing. We also describe strategies to predict and validate neoantigens from RNA sequencing data.

## Introduction

Since the immune system recognizes tumor-specific peptides, they are potential targets for personalized neoantigen-based immunotherapy. They are presented by the major histocompatibility complex (MHC) on the surface of tumor cells, which can be recognized and killed by T cells. These antigenic peptides are largely classified into three groups: viral antigens, cancer germline antigens, and mutation-derived neoantigens [1]. Among them, mutation-derived neoantigens have been focused in cancer genomics because the causal role of somatic mutations in cancer have been well-studied. Indeed, the mutation burden of a tumor is known to be correlated with its response to immunotherapy [2]. However, exome analysis–based immunotherapy strategies have limitations, since they can identify only neoantigen candidates occurring at the DNA level [3]. This suggest that a deeper understanding of the generation of neoantigens and better strategies to identify them will be required to improve neoantigen-based immunotherapy.

Recent progress in sequencing technologies has enabled the profiling of RNA processing events caused by various forms of post-transcriptional regulation. In particular, alternative splicing and RNA editing have drawn considerable attention since they promote proteome diversity through changes in amino acid sequences. In addition, the transcript isoforms are easily detectable utilizing conventional RNA sequencing (RNA-Seq) data if proper computer-based methods are applied. Accordingly, analyses of large-scale RNA-Seq data have shown the genome-wide prevalence and regulation of alternative splicing and RNA editing [4,5]. Data sources such as The Cancer Genome Atlas (TCGA) and the Genotype-Tissue Expression (GTEx) project have enabled systematic investigations of their association with cancer. Investigators have shown that these RNA processing events are significantly more frequent in cancer samples than in matched normal samples, contributing to antigenic diversity [6,7].

**Fig. 1.**
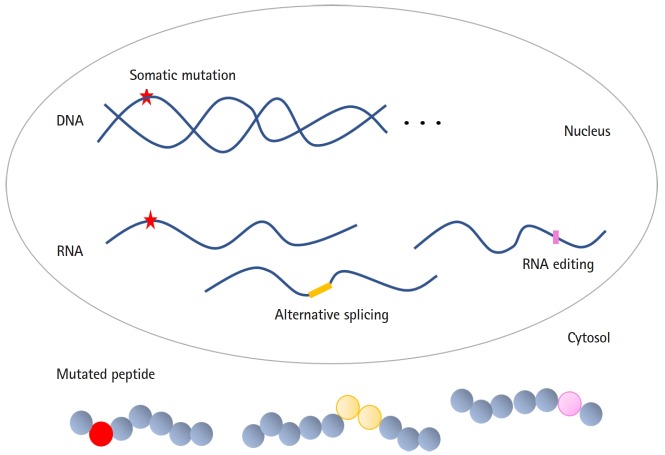
Schematic presentation of various sources of neoantigens. Somatic mutations in DNA (red star), alternative splicing (yellow bar), and RNA editing (pink bar) can introduce significant modifications of DNA or RNA molecules. Various regulation steps can induce sequence changes in the final gene products, and the resulting mutated peptides can be presented on MHC. Alternative splicing occurs in the cell nucleus, but RNA editing can be performed in the nucleus and cytosol, as well as within mitochondria. The colored circles shown in the cytosol indicate amino acids changed by genetic events.

Of note, like somatic mutations, alternative splicing and RNA editing can produce cancer-specific antigens (Fig. 1). Genetic alterations (marked by an orange or pink color in the figure) can occur after transcription. As a result, unlike somatic mutations, RNA-level genomic changes are not preserved in offspring cells. Another noteworthy difference is that the altered transcripts have a wide range of expression levels depending on environmental conditions. Therefore, alternative splicing and RNA editing can be quantified using numerical values ranging from 0% to 100%, indicating the need for user-defined thresholds for modification calling. Recent studies have reported that cancer-specific RNA processing can be a source of immunogenic epitopes [8,9]. In this review, we outline recent work involving the large-scale screening of neoantigens produced by alternative splicing and RNA editing. We also describe analytical strategies to predict and validate neoantigens from RNA-Seq data.

## Alternative Splicing

**Fig. 2.**
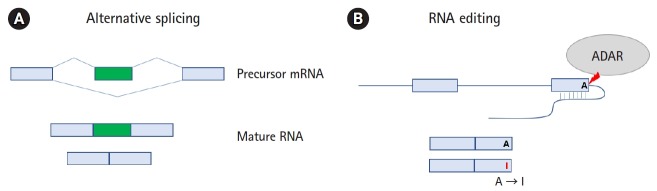
Examples of RNA processing steps to produce two RNA isoforms. Two different mRNAs are produced from alternative splicing (A) and RNA editing (B). The top panel shows precursor mRNAs and the bottom shows mature RNAs after posttranscriptional processing. Exons are illustrated as boxes, while lines represent introns. For alternative splicing (A), exon skipping is shown as one example of the numerous modes. In this case, an exon is selectively included from the primary transcript. Constitutively expressed exons are depicted in green, and alternatively spliced exons are depicted in light blue. For RNA editing (B), the conversion of adenosine (A) to inosine (I) is shown in the double-stranded RNA structure. The editing is catalyzed by the adenosine deaminase acting on RNA (ADAR) enzyme. Most events occur in the noncoding region of the transcript, but the minor case showing editing in a coding sequence is shown in this figure.

Alternative splicing can produce multiple transcripts according to the patterns through which exons and introns are joined (Fig. 2A). Alternative splicing is known to affect more than 90% of multi-exon human genes [10]. The basic patterns include exon skipping, the use of alternative 5' or 3' splice sites, mutually exclusive exons, and intron retention. Exon skipping is the most common type of alternative splicing in animals, whereas intron retention is the least prevalent form [11]. The functional consequences of alternative splicing can be predicted using various annotation data, with possibilities including the gain/loss of protein domains, signal peptides, and coding potential [12,13]. Intron retention often leads to nonsense-mediated decay (NMD) by introducing a premature termination codon, resulting in reduced gene expression [14]. These RNA-level changes can be further propagated to proteomic changes through their effects on protein-protein interaction [15]. Numerous computational methods have been developed to identify regulated splicing events in RNA-Seq data and to predict their upstream regulators in a genome-wide manner [16]. Bioinformatics tools are largely classified into two groups: tools to examine known events and tools capable of detecting novel events. The latter tools are useful for uncovering unannotated cancer-specific events, but their algorithms are complicated, and the running time is generally longer.

Numerous studies have revealed cancer-specific splicing changes, suggesting their applicability for cancer diagnosis and therapy [17-19]. Recent pan-cancer analyses using TCGA data identified that alternative splicing events are indeed frequently altered in cancer and some of them can contribute to the oncogenic process. The altered events take place through a variety of *cis*- and *trans*-acting regulatory factors. Aberrant splicing patterns can be induced by genetic variants in splicing regulatory sequences around splice sites. The variants near splice sites are more likely to have functional effects on splicing by disrupting existing splice sites or creating new sites [20,21]. In addition to variants that directly change splice sites, common variants throughout the genome can be associated with the splicing phenotype. These are termed splicing quantitative trait loci (sQTL), and they can be analyzed by integrating population-scale genotype and RNA-Seq data [22,23]. Lastly, *trans*-acting splicing regulators can activate or repress splicing of their target exons [24].

With growing interest in cancer immunotherapy, two recent studies evaluated the contributions of alternative splicing to neoantigen production. The first study performed a comprehensive analysis of alternative splicing with the development of an integrated workflow utilizing large-scale genomics datasets [7]. Kahles et al. [25] identified quantitative differences in splicing between cancer and normal samples, and their enrichment in splicing categories also differed. For example, splicing regulation in cancer samples was enhanced in the categories of alternative 3' splice sites and mutually exclusive exons. That research group previously developed the SplAdder toolkit to identify novel splicing events with good performance in a large population [25]. Using this tool, they identified 251,000 novel exon-exon junctions (referred to as neojunctions) with an average of 930 per cancer sample. The cancer-specific junctions should be absent in the GTEx reference data, since they are rarely expressed in normal reference samples [7]. A proteomics database, containing Clinical Proteomic Tumor Analysis Consortium (CPTAC) mass spectrometry data, was used to confirm the expression of peptides derived from alternative splicing. An important finding was that neoantigens derived from alternative splicing events were more frequent than single-nucleotide variants, even though the former category had relatively low levels of expression. Lastly, the study pointed out that their predictions of neoantigens were not complete for several reasons, including the fact that they excluded intron retention events from neoantigen prediction.

In this context, a recent study was meaningful because it focused on intron retention, a splicing type that was neglected in the previous study. Intron-containing peptides are often subject to degradation by NMD, and the cleaved peptides can be presented on MHC class I molecules. Smart et al. [26] developed a computational strategy to detect intron retention events from RNA-Seq data. Using two cohorts of melanoma patient data with checkpoint inhibitor treatment, they found that intron retention was an important mechanism for neoantigen prediction that should be checked, because as many intron-retaining neoantigens as somatic mutation–derived neoantigens were present in most patient samples. However, they were not associated with the clinical outcomes of checkpoint inhibitor therapy, unlike somatic mutation–derived neoantigens. Taken together, the above two studies significantly expanded the boundaries of neoantigen prediction by considering both DNA and RNA alterations.

## RNA Editing

RNA modifications change the biochemical composition of RNA molecules and affect their structure and metabolism. RNA modifications are called the “epitranscriptome,” meaning that they are forms of post-transcriptional alterations that do not affect the RNA sequence, in analogy to how the term “epigenome” describes functionally relevant changes that do not involve changes in the DNA sequence [27]. Recent studies have revealed that many of these modifications are remarkably widespread across the genome, evolutionarily conserved, and functionally important. More than 100 distinct types of RNA modifications have been identified. Most RNA modifications do not change nucleotides, but RNA editing is accompanied by a change in the RNA sequence itself. One of the common examples is the deamination of adenosine (A) to inosine (I), which is recognized as guanosine (G) in RNA sequencing. The A-to-I editing is mediated by the adenosine deaminases acting on RNA (ADARs) protein family, which prefers double-stranded RNA structure [28] (Fig. 2B). The edited sites are mostly found in noncoding regions of RNA, which may have regulatory potential. RNA editing of the protein-coding sequence can result in the expression of functionally altered proteins. In addition, the editing can have an impact on RNA splicing, indicating the interplay of these mechanisms [29]. The biological consequences of RNA editing are broadly linked to RNA metabolism and function, including mRNA stability, splicing, nuclear export, and localization [30].

As mentioned, A-to-I editing is detectable from RNA-Seq data by modifying the analysis pipeline. A prediction should be carefully made due to frequent false positives arising from single-nucleotide polymorphisms or sequencing errors. This limitation has been overcome in recent years by developing bioinformatics methods for accurate predictions. By comparing genomic DNA and RNA sequencing data from the same individuals, the biological noise from genomic polymorphisms could be reduced. Technical noise caused by sequencing errors and incorrect alignment can be removed by focusing on high-quality reads. Public databases collecting well-annotated edited sites are also useful for reliable screening and functional annotation [31,32]. In the near future, new platforms such as nanopore sequencing will solve current technical hurdles by allowing direct detection of editing in full-length RNA molecules [33].

Recent advances in RNA editing research have contributed to scientists’ understanding of the mechanisms involved in human cancers through the accumulation of evidence of mutated peptides induced by RNA editing [34,35]. Through an integrated analysis of TCGA genomic data and CPTAC proteomic data, Peng et al. showed that A-to-I RNA editing made a notable contribution to increased protein diversity in human cancers [36]. According to their analysis, a considerable number of editing events lead to changes in the amino acid sequence, indicating the possibility that A-to-I editing may be a source of producing mutated peptides. More direct evidence of RNA editing for neoantigen production was reported in recent research by Zhang et al. [6]. Using proteogenomics screening, they identified five edited peptides and confirmed their tumor association and their immunogenicity regarding T cell recognition and killing. In addition, they showed experimental evidence responsible for the biological mechanism of RNA editing depending on ADAR expression. In addition to cancer research, Roth et al.[37] provided evidence that RNA editing is significantly increased in systemic lupus erythematosus patients, promoting autoimmune progression by increasing the autoantigenic load.

## Conclusion

Improvement of neoantigen prediction from patient samples is an important issue for developing effective immunotherapy. Current approaches to neoantigen prediction have focused on somatic mutations, even though genetic changes affecting protein production can occur at many different levels. Most computational tools developed so far have aimed to evaluate the effect of non-synonymous DNA variants on producing mutated peptides. Representative analysis pipelines such as pVAC-Seq [38] and Neopepsee [39] have been established for somatic mutation analysis.

The recent studies described herein suggest that alternative splicing and RNA editing can serve as important sources of neoantigens. The challenge in utilizing RNA-derived neoantigens is the development of bioinformatics methods with increased accuracy and performance. RNA-Seq is now a popular technique, and data on RNA-Seq have been accumulating on a daily basis. However, many researchers feel that it is not easy to detect posttranscriptional modifications, such as alternative splicing and RNA editing. Therefore, improvement of the analysis pipeline will be required to make the RNA-derived neoantigen prediction more reliable.
